# Biden’s Security Policy: Democratic Security or Democratic Exceptionalism?

**DOI:** 10.1007/s10272-021-0945-1

**Published:** 2021-01-26

**Authors:** Simona R. Soare

**Affiliations:** European Union Institute for Security Studies, 100 Avenue de Suffren, 75015 Paris, France

## Abstract

By emphasising the internal-external security nexus inherent in democratic security, the US could aspire again to lead through the example of its democracy’s resilience and ability to self-correct.

Can the Biden administration chart a new course for American security policy given the unprecedented level of polarisation and social unrest in the United States? Can the US lead internationally and build a new transatlantic partnership while tackling the layered political, economic and health crises it faces at home? This article examines the Biden administration's security priorities, tracing elements of change and continuity in US foreign and security policy, and the challenges it faces as it tries to reassert US power and leadership, reassure allies and rebuild partnerships.

This paper argues that President Biden's foreign and security policy should build on democratic security by offering the prospect and promise of a fresh democratic future, not merely a fixed version of the past, while avoiding the pitfalls of democratic exceptionalism. This is a daunting task, though there is no incompatibility between international leadership and rebuilding democracy, the economy and resilience internally. By emphasising the internal-external security nexus inherent in democratic security, the

* The author writes in a personal capacity. The ideas reflected in this article do not reflect the views or policies of the EUISS or the EU more broadly.

US could aspire again to lead through the example of its democracy's resilience and ability to self-correct. This involves rethinking how the US exercises power and leadership in the context of an unprecedented level of fragility in the American political system and significant economic challenges in the aftermath of the COVID-19 pandemic.

The article proceeds in three sections. First, it outlines the Biden administration's short- and long-term security priorities and highlights the elements of change and continuity compared to the administrations of his two predecessors, Donald Trump and Barrack Obama. The second part examines the changing US view of strategic partnerships and reflects on the pivotal role of the transatlantic partnership, including the strategic US-EU relationship, in US foreign and security policy. The final part of the paper highlights the domestic and international challenges the US will face in implementing President Biden's democracy agenda in foreign and security policy.

## Biden's security priorities and democratic security

The task before the Biden administration is monumental. The credibility, reliability and legitimacy of the US as a world leader, as well as its ties to Europe, its closest ally and partner, have been eroded by a toxic Trump presidency. Meanwhile, President Biden and his team inherit both a world and a country that are decidedly more polarised, unsettled, rapidly changing and fraught with uncertainty and complex security problems competing for their attention, and they will have less resources and less time to address them.

Joe Biden first outlined his foreign and security policy vision in a 2020 Foreign Affairs article: rebuild the foundations of American power (economy, socio-economic equality, innovation, democracy), restore American democracy and revitalise international partnerships (Biden, 2020a) through a combination of democratic security, reformed multilateralism, new liberal institutionalism and multi-stakeholder engagement on issues that cut across the internal-external security nexus. This vision, which Biden called "a foreign policy for the middle class", acknowledges the connections between economic displacement and pressures at home and the US role in the world. It articulates a multilateralist approach to managing the shifting global balance of power, emerging technologies and globalisation – or a new liberal internationalism (Blinken, 2016). And it attempts to reconcile domestic democratic, economic and societal security with due considerations to security at the individual, national and international levels – which international relations literature calls democratic security (Steuer, 2019). In short, to rebuild democracy at home, the US has to strengthen democracy abroad (Wright, 2021).

President Biden's vision, though, goes further and touts a return of American democratic exceptionalism and a self-perception of the US as the "beacon" of democracy and the indispensable leader of the international system. In President Biden's words: "It falls to the United States to lead the way. No other nation has that capacity. No other nation is built on that [democratic] idea" (Biden, 2020a). Opinion polls (see Figure 1) and experts challenge this reaffirmation of American exceptionalism: "the question for the incoming Biden administration is whether the rhetoric of American exceptionalism has purchase in a world where (…) the 'city on a hill' story does not shine as it once did" for domestic and international audiences (van Engen, 2021). Others argue Biden's international security agenda is disconnected from American domestic realities of unprecedented political unrest and disregard for the rule of law, and they question whether the US has the legitimacy, resources and strategic attention necessary to lead internationally while confronting domestic challenges (Ashford, 2021). Following four years of Trump's disengagement from the multilateral international system and assault on rule of law (Hill, 2021), Biden's vision for America's "foreign policy for the middle class" (Biden, 2020a) approximates democratic security, but for domestic reasons risks coming too close to an exercise in democratic exceptionalism.

## Biden's approach to geopolitics and globalisation

The challenges to US national security posed by China, Russia, Iran and North Korea as well as international terrorism will remain top concerns under the Biden administration. This is a strong element of continuity with the Trump administration's 2017 shift to great power competition. However, the Biden administration is expected to adopt a less unilaterally confrontational tone in relations with China, while being more hawkish in its relations with Russia. In 2020, answering *New York Times*' questions about his foreign policy priorities, candidate Biden pledged to re-emphasise a two-state solution in the Israeli-Palestinian conflict but considered the return of the US embassy to Tel Aviv unnecessary (Goldberg, 2020). Much like the Obama-Biden administration, the Biden-Harris administration is expected to champion multilateral efforts at nuclear non-proliferation. Iran and North Korea will take priority, albeit under a different approach: candidate Biden confirmed that he intends to rejoin the Joint Comprehensive Plan of Action (JCPOA) should Tehran remain in compliance (Biden, 2020b), and despite Iranian provocations (Rassmussen and Norman, 2021), his administration might still pursue this goal. Summit diplomacy with North Korea will likely be replaced by a return to a multilateral diplomatic effort at denuclearisation. Like his two predecessors, the Biden-Harris administration has committed to ending the "forever wars" of the Middle

East and replacing them with a small military footprint for counterterrorism purposes (Biden, 2020a).

In addition to these elements of geopolitical continuity, the administration will also emphasise a set of transnational, globalised security challenges, including tackling the global health crisis and the COVID-19 pandemic, climate change, socio-economic inequality, reforming the international trade system, the governance of emerging technologies consistent with liberal values and curbing irregular migration. In addition to the challenges of negotiating common positions with allies and partners, the historical American partisan divide (Figure 1) on these foreign policy priorities is already a significant challenge to the success of Biden's security policy (Walt, 2019).

A flagship initiative for the Biden administration in its first year in office will be the Summit for Democracy, an initiative meant "to put strengthening democracy back on the global agenda" (Biden, 2020a) and build resilience against the relentless assault from authoritarianism, nationalism, populism and corruption. It is less clear how the new administration will bring this new platform to bear in relation to other strategic priorities, particularly trade, technology and defence, but the effort to integrate these four dimensions into a new strategic approach to multilateral and bilateral partnerships is evident in Washington.

More broadly, Biden's team will emphasise human rights and democratic principles and shift from a transactional to a more traditionally conditional US pressure on partners. This could take the form of leveraging US strategic relations with challenging allies like Poland, Hungary and Turkey to achieve concessions on respect for the rule of law and human rights (Harvard-DGAP, 2020, 4). However, following the 6 January 2021 storming of the US Capitol by pro-Trump rioters, experts argue: "The power of America's example will be dimmer than it once was; American arguments will be harder to hear" (Applebaum, 2021). Some have called on President Biden to abandon the idea of an international Summit for Democracy and instead focus inwards on American democratic renewal e.g. (Goldgeier and Jentlesen, 2021).

The first short-term step for the Biden administration is to re-engage the US in the international system and to reaffirm the strength of enduring US alliances and partnerships eroded or undermined by President Trump's foreign policy through symbolic diplomatic gestures. These will be a priority for the administration in the first three to six months in office, depending on the evolution of the COVID-19 epidemic in the US. American officials recognise this should include high-level visits to major European capitals, to the EU and NATO, rejoining the Paris climate accord, the

Figure 1

**Americans divided on US foreign and security challenges (2018-2020)**

% who say it should be a top foreign policy priority

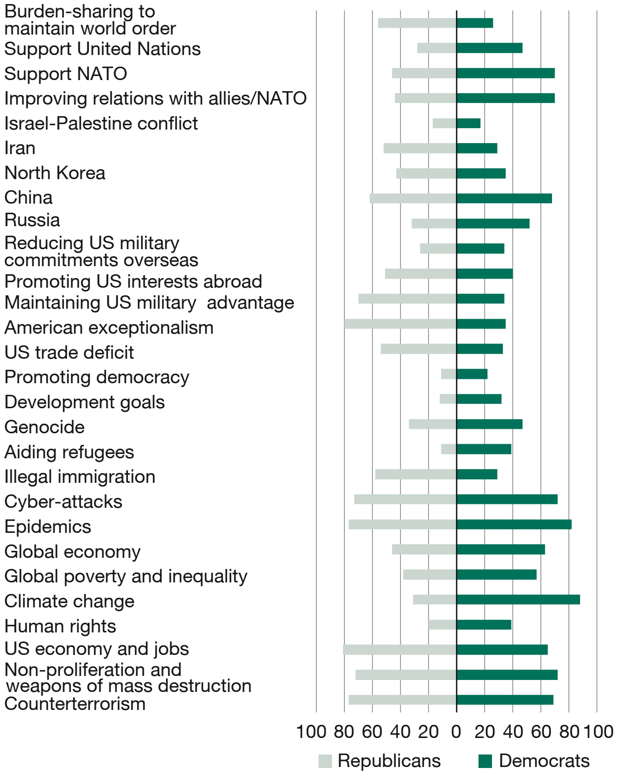

Sources: PEW Research Center, 2018-2020; Chicago Council on Global Affairs.

JCPOA and the World Health Organization, joining the COVAX initiative and extending the New START agreement with Russia. Other Biden security priorities will take longer and will be far more challenging to realise. Building an international democratic agreement on a coordinated policy towards China, negotiating a plan to reform the World Trade Organization to reflect a fair and level playing field, agreeing on a common democratic approach to global governance and regulatory and standardisation issues in emerging technologies will take time and require a significant multilateral effort.

## Biden's layered approach: What role does the transatlantic partnership play?

President Biden and his team are strong supporters of the transatlantic partnership, but the new administration will espouse a new and more strategic approach to partnerships and alliances, including in relation to NATO and the EU. Three pillars will structure American efforts in the next four years.

### Pillar I: NATO adaptation

The first pillar is to regain trust and reaffirm the unbreakable security and defence ties within NATO. The reason behind this is strategic – the Biden administration correctly regards the North Atlantic alliance as a key tool in confronting and containing aggressive Russian behaviour and Chinese influence. The Alliance is already undergoing a detailed self-reflection process, i.e. NATO 2030, after which Secretary General Stoltenberg will propose that allies develop a new NATO Strategic Concept in early 2021. This represents an opportunity for the US to exercise more prominent NATO leadership right from the start of the new administration. The upcoming 2021 NATO Summit will open diplomatic doors to allow for symbolic gestures of reaffirmation of the American commitment to European security. It will equally be a useful framework for substantive discussions about the future direction of the Alliance on emerging technologies, climate change, China, critical infrastructure protection as well as how to deal with difficult allies that transgress on democratic principles. In this context, the Biden administration could drive a new stage of NATO adaptation beginning in 2021: “As president, I will do more than just restore our historic partnerships; I will lead the effort to reimagine them for the world we face today” (Biden, 2020a).

The NATO 2021 Summit will also offer an excellent opportunity for the Biden administration to lay out a new approach to transatlantic burden-sharing. The Biden administration’s qualitative and quantitative understanding of European contributions to transatlantic burden-sharing is expected to be more flexible and more encompassing than that of the Trump administration but continued American pressure on European investment in defense will remain a US policy staple. According to NATO, since 2014 Europeans have added over €100 billion to defence spending, though experts warn the COVID-19 economic recession could lead to defence budget cuts. While this administration will likely reverse some announced US troop withdrawals from Europe (Deni, 2020, 38), the overall shift in American forward military presence on the continent and towards the Eastern flank (especially the Baltic and Black Seas) is expected to continue. In addition, the US drive towards the accelerated adoption of emerging technologies in defence applications could mean American pressure on European allies to invest in defence innovation efforts unilaterally or collectively and to progress rapidly on military information and data sharing (Soare, 2020a).

### Pillar II: Enhanced US-EU cooperation

The Biden administration’s understanding of transatlantic burden-sharing could feature a re-evaluation of the role of and relations with the EU. Not all members of the Biden administration are uniform in their view of European strategic autonomy and there is still a healthy dose of scepticism about European relations with China, EU defence initiatives, digital taxation and more. The Biden administration will be less confrontational and will cautiously encourage European security efforts, while maintaining the strict conditionality policy around NATO-EU cooperation (Soare, 2020b, 51). Challenging transatlantic differences, like taxation, trade and digital content regulation will entail hard conversations between Washington, Brussels and European capitals before American concerns for fair trade and taxation practices and European aspirations for technological sovereignty and strategic autonomy can be accommodated. Nevertheless, it is noteworthy that members of the administration contend that the US needs to change course and engage with the EU in light of its regulatory powers on priority files like trade, technology, industrial policy, security and climate (Soare, 2020b, 53; Bergmann, 2021).

Remarkably, the European Commission and the EU’s High Representative Josep Borrell have been proactive in shaping a New Transatlantic Agenda that underlines the interest in working closer on trade, labour, climate, science and technology, regulatory matters (digital and carbon taxes, digital services and platforms) and more. Several cooperation mechanisms are under discussion, including the creation of an EU-US Trade and Technology Council, a high-level dialogue on China, a Dedicated Dialogue on Security and Defence, a transatlantic artificial intelligence agreement (European Commission, 2020). While further clarifications are needed, there is great commonality between Biden’s “foreign policy for the middle class” and similar EU interests, potentially providing a basis for a much broader cooperation on security-relevant, albeit not defence specific, areas (CSIS, 2021). Democracy is a common concern and the European Commission endorsed Biden’s Summit of Democracies proposal, but Europeans are still apprehensive about entering a full-fledged ideological competition with China and Russia.

Overlapping security concerns and Biden’s change of course away from unpopular Trump policies (Figure 2) create the prospects of closer EU-US cooperation. A proposed EU-US Summit in the first half of 2021, along with a number of expected high-level US visits to main European capitals will provide ample opportunities for the new US administration to lay out its vision of relations with the EU and articulate differences of opinion on key issues such as China, digital policies, taxation and trade. European capitals will expect the Biden White House to accommodate a sense of shared US-EU leadership in managing global issues like climate, trade, taxation and

Figure 2
**European disapproval**
rate of approval/disapproval, 2017-2019, %

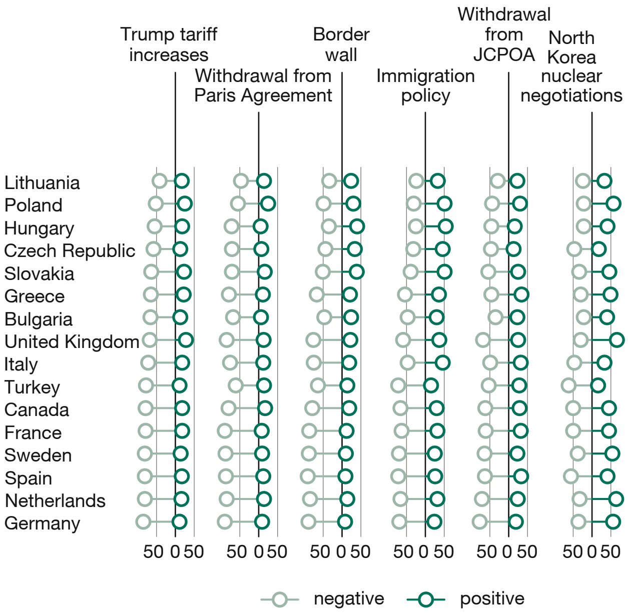

Notes: Europeans strongly disapprove of the way the US has handled the COVID-19 crisis (85%), of American withdrawal from the Paris Agreement on climate change (75%) and of the imposition of US tariffs on Europe (69%). European majorities also disagree with American policies on immigration and the border wall as well as with the US withdrawal from the Iran nuclear deal.

Sources: PEW Research Center, 2019, 2020; Dalia Research, 2020.

technologies. Early American commitment to embedding the transatlantic partnership at the core of this global effort to manage the forces of international change (Soare, 2020, 5) would be most welcome in Europe.

## Pillar III: European and Asian partnerships in US security policy

A third aspect of President Biden's view of international partnerships is the effort to bring together European and Indo-Pacific partners, like-minded democratic countries that share a concern for the rise of authoritarianism. This effort goes beyond the Summit for Democracy or the US effort to steer NATO towards confronting the Chinese challenge – in Europe – and developing closer cooperation with global partners, including in the Indo-Pacific (Burton, 2020).

The US has encouraged the EU and European states to increase engagement with Asian partners such as Japan, South Korea and Australia, and Europeans are currently developing their common approach to the Indo-Pacific. Given that Washington has long preferred a limited European role in Indo-Pacific strategic affairs (Bacon, 2020, 94-95), it remains unclear whether the Biden administration will change course and, if so, to what degree European involvement in Indo-Pacific security is possible.

Other differences among the European and American views on how to embed transatlantic relations at the centre of broader multilateral efforts focus on challenging interdependencies closer to home. One such difference is the European view that the EU-US relationship should be a second transatlantic pillar, in addition to NATO (Biscop, 2020), whereas US experts have expressed an interest in a broader format that brings together the US, the EU, Canada, the UK (Harvard-DGAP, 2020) and, possibly, Indo-Pacific partners. The details of such cooperative frameworks are still unclear, though the new US administration will be keen to encourage closer UK cooperation with the EU and European partners in the aftermath of Brexit. At the same time, there are worries that Washington could leverage the common views among Ottawa, London and Washington regarding EU defence, strategic autonomy and technological sovereignty in future negotiations.

## Biden's challenges

It is too early to determine President Biden's chances of success as many security issues will require far more than a four-year presidential term to solve. In many ways, and perhaps unfairly, this administration's success will be defined not just by the policies it adopts, but by how it is able to sustainably restore American credibility, decisively re-engage the US in the multilateral rules-based international order and firmly set US foreign and security policy on a new course for the foreseeable future.

This is easier said than done under the current domestic and international circumstances. The president and his team have the advantage of Democratic control of the White House and Congress for at least the next two years. This widens the president's freedom of manoeuver – at least temporarily. On the other hand, both the Biden administration and the Democratic Party will be hard pressed to take effective measures to ensure Trumpism does not return in the next two to four years – a significant concern of European and Asian partners.

President Biden has called on Congress to focus on social and economic recovery measures rather than the historical second impeachment of President Trump. The challenges of managing the COVID-19 epidemic in the US, where the death rate on 14 January reached an eye-widening 42,000 deaths in one day, and putting the US

economy back on track, even with the implementation of the new $1.9 trillion "American Rescue Plan" (Smialek, 2021), are huge and will likely command this administration's strategic attention for the better part of 2021.

Political polarisation in the US has reached worrying levels and culminated in the storming of the US Capitol by a group of rioters who attempted to halt the congressional confirmation of the election of Joe Biden as the next US president (Baker, 2021). The intensity and frequency of the domestic challenges to the American rule of law and democratic institutions are unprecedented in the last half a century or more – a cause for concern in Europe (Schemm et al., 2021). Several sources warn this social unrest is just the beginning and that more violent protests driven by social and political polarisation may follow (Sargent, 2021). Convincing the American middle class of the virtues of international liberal institutionalism poses significant challenges (Ahmet et al, 2020). Moreover, it will be equally challenging for Biden to accommodate the progressive wing of the Democratic Party's strong preferences on domestic, foreign and security policy.

The events of 6 January in Washington drew considerable internal and international criticism over the legitimacy of the US to promote a democratic security agenda seeing as its own house was not in order. "America has to be a functioning democracy before it can be an exemplary one" (Goldberg, 2020) and "ambitious foreign-policy goals are completely out of step with the realities of the country's domestic political and economic dysfunction" (Ashford, 2021), the argument goes. American experts are not wrong in saying that American democracy is tested at home, but democracy is now tested everywhere. If the age of American leadership based on the power of its democratic example is over, then Washington needs to adapt and rethink its leadership in a world of post-US dominance. There is just as much merit in America leading through the example of its democracy's resilience and ability to self-correct, regardless of how messy it is now. The power of this American example – and the US opportunity to still lead the world towards democratic security and resilience – should not be squandered when virtually every democracy in the international system is facing similarly strong illiberal forces. However, steering US security policy firmly towards democratic security and away from democratic exceptionalism will be essential to President Biden's success, particularly in relation to Europe.
